# Are Lipases Still Important Biocatalysts? A Study of Scientific Publications and Patents for Technological Forecasting

**DOI:** 10.1371/journal.pone.0131624

**Published:** 2015-06-25

**Authors:** Karina de Godoy Daiha, Renata Angeli, Sabrina Dias de Oliveira, Rodrigo Volcan Almeida

**Affiliations:** 1 Departamento de Bioquímica, Universidade Federal do Rio de Janeiro, Rio de Janeiro, Brasil; 2 Unidade de Biologia, Centro Universitário Estadual da Zona Oeste, Rio de Janeiro, Brasil; 3 Agência de Inovação, Universidade Federal do Rio de Janeiro, Rio de Janeiro, Brasil; Wageningen University, NETHERLANDS

## Abstract

The great potential of lipases is known since 1930 when the work of J. B. S. Haldane was published. After eighty-five years of studies and developments, are lipases still important biocatalysts? For answering this question the present work investigated the technological development of four important industrial sectors where lipases are applied: production of detergent formulations; organic synthesis, focusing on kinetic resolution, production of biodiesel, and production of food and feed products. The analysis was made based on research publications and patent applications, working as scientific and technological indicators, respectively. Their evolution, interaction, the major players of each sector and the main subject matters disclosed in patent documents were discussed. Applying the concept of technology life cycle, S-curves were built by plotting cumulative patent data over time to monitor the attractiveness of each technology for investment. The results lead to a conclusion that the use of lipases as biocatalysts is still a relevant topic for the industrial sector, but developments are still needed for lipase biocatalysis to reach its full potential, which are expected to be achieved within the third, and present, wave of biocatalysis.

## Introduction

“*The possible substrates for lipase are to be numbered in millions*”, wrote the eminent biochemist J. B. S. Haldane in his monograph entitled “Enzymes”, in 1930 [1]. Lipases were firstly observed in the XIX century [2], and have kept researchers' attention since then. Such success lies in the characteristics of this class of enzymes. The triacylglycerol acyl hydrolases (E.C. 3.1.1.3) hydrolyze carboxylic ester bonds, releasing carboxylic acids and alcohols. Under low water conditions, however, reactions known as esterification or transesterification reactions may occur [3]-[4]. Their capacity of catalyzing such reactions with high efficiency and stability, their chemo-, regio- and enantioselectivity, as well as not requiring cofactors and being active in organic solvents render lipases very attractive and versatile enzymes from the industrial point of view [2], [5].

Lipases may be applied in various types of industries, historically being one of the most important groups of biocatalysts for biotechnological applications [6]. The most commercially important application for lipases is in detergent industry, closely followed by the food & beverage industry. Lipases find several uses in food technology, such as in flavor development, baked foods, manufacturing of dairy products, production of butter and milk equivalents, processing of meat and fish, animal feed, and many other applications [7]. Another important use of these enzymes is as biocatalysts in the resolution of racemic mixtures for producing pure enantiomers for the pharmaceutical industry [4]. The Thalidomide tragedy in the late 1950s is one emblematic case that led to more severe rules and increased pressure for enantiomerically pure compounds [8]. Although biocatalysts are employed in industrial processes for chemical synthesis, enzymes used for this purposes may be considered specialty enzymes as they are required in small quantities and have a higher value than industrial enzymes [9]. The use of lipases for the production of biodiesel, which has been considered an alternative renewable and eco-friendly fuel, has grown in importance in the last years [10]. Enzymatic transesterification has potential to substitute the most often industrially used method–the alkaline transesterification, as it has several advantages in relation to the latter, such as the esterification of both free fatty acids and triglycerides in one step without the need of a subsequent washing step [11].

Almost ten years ago, Jaeger and Eggert asked “*What is it that makes lipases so attractive*?*”* [6] Now, we reformulate such question to *“Are lipases still attractive*?*”* Aiming at answering this question, the present study investigates the technological development of four strategic industrial sectors at different stages of development and with different technological levels. By analyzing publications and patents as scientific and technological indicators, respectively, this work provides an overview of the R&D efforts in these sectors over a thirty year period. Finally, cumulative patent data are used for analyzing the life cycle of such technologies and for technological forecasting.

## Methodology

### Patent Search

The searches for patent documents were carried out in July 2014 and April 2015 at Orbit.com, a private patent search portal covering more than 100 patent authorities. A specific search strategy was set up for each of the industrial applications of lipase analyzed, i.e., resolution of racemic mixtures, and production of detergents, biodiesel and food and feed products (Table 1). The strategies were based on the combination of keywords in title and abstract of the patent documents, classes and subclasses of the International Patent Classification (IPC) and first priority date of the patent family. The documents found in the searches had their title and abstract analyzed in order to exclude those that were not related to each of the search scopes.

**Table 1:** Patent search strategies set up for each of the four applications of lipase.

Lipase Application	Search Strategy
Kinetic Resolution	((((LIPASE?)/BI AND (ENANTIO+ OR STEREO_SELECTIV+ OR (OPTIC+ W (ACTIV+ OR PUR+ OR ISOMER+)) OR RESOLUTION OR ((SPLIT+ OR SEPARAT+ OR RESOLV+ OR ESTERIF+) S (CHIRAL+ OR RACEM+)))/BI AND (C12P OR C07C OR C07D OR C12R OR C12N)/IC) NOT (LIPASE S (INHIBITOR+ OR MODULAT+ OR DEFICIENCY))/BI)) AND (EPRD = 1983-01-01:2012-12-31)
Production of Detergents	(((LIPASE?)/BI AND (DETERGENT? OR +WASH+ OR LAUND+ OR BLEACH+ OR CLEAN+ OR SOAP+)/BI) AND (C11D OR C12N)/IC) AND (EPRD = 1983-01-01:2012-12-31)
Production of Food and Feed Products	((((LIPASE?)/BI AND (FOOD+ OR FEED+ OR JUICE OR BEVERAGE+ OR FISH OR DOUGH OR MILK+ OR CHEESE+ OR BUTTER+ OR MEAT OR EGG OR YOLK OR BREAD))/BI) NOT ((INHIBITOR+) S LIPASE/BI)) AND (A23+ OR A21+ OR C12N)/IPC AND (EPRD = 1983-01-01:2012-12-31)
Production of Biodiesel	(((LIPASE?)/BI AND ((+FUEL? OR +DIESEL?) OR (FATTY ACID W (METHYL OR ALKYL) W ESTER?) OR FAME OR FAAE)/BI) AND (C12P OR C10L OR C12N OR C11C OR C10G)/IC) AND (EPRD = 1983-01-01:2012-12-31)

“?” is a truncation symbol replacing zero or one character; “+” is a truncation symbol representing any number of characters; “_” is an operator indicating that two terms may be connected in a single word or separated in two adjacent words; “W” is an operator indicating that the adjacent terms are in the order specified; “S” is an operator indicating that the terms are in the same sequence. /BI: the terms were searched in the title and abstract of the patent documents; /IC: international patent classification; EPRD: first priority date of the patent family.

### Scientific Publication Search

The searches for scientific publications were carried out in September 2014 and April 2015 at Web of Science, a research platform maintained by Thomson Reuters covering more than 12,000 journals in all subject areas. A specific search strategy was set up for each of the industrial applications of lipase analyzed, i.e., resolution of racemic mixtures, and production of detergents, biodiesel and food and feed products (Table 2). The strategies were based on the use of keywords in the title and topic, and publication year of the documents. The publications found in the searches had their title and abstract analyzed in order to exclude those that were not related to each of the search fields.

**Table 2:** Publication search strategies set up for each of the four applications of lipase.

Lipase Application	Search Strategy
Kinetic Resolution	Title:(lipase$) AND topic((kinetic or chiral or lipase or hydrolysis or enzymatic) near/1 resolution) and publication years:(1983–2012)
Production of Detergents	TITLE:(lipase$) AND topic(*detergent$ or laundr* or dish*or ((remov* or clean* or wash*) near/1 (stain* or oil$ or grease or fat* or triglyceride$or soil$))) and publication years:(1983–2012)
Production of Food and Feed Products	TOPIC:(lipase* and (((food* or feed) near/5 (industr* or formulation* or product*)) or (cheese* near/1 (ripening or flavor* or making or product*)) or ((butter or milk) near/0 (equivalent* or substitute*)) or “dairy product*” or dough*)) AND PUBLICATION YEARs:(1983–2012)
Production of Biodiesel	Title:(lipase$) AND topic(biodiesel or diesel or biofuel$ or fuel$) and publication years:(1983–2012)

“*”is a truncation symbol replacing any number of characters

“$” is a truncation symbol replacing zero or one character; “NEAR/n” is an operator indicating that two terms are n words distant from each other.

### Logistic Curves

Cumulative patent data found for each of the industrial sectors were fitted into S-shaped curves by using Loglet Lab 2 Software, which was developed by the Rockefeller University. The software was supplied with the patent data found in the searches and it was fitted to logistic, in the case of biodiesel application, and bi-logistic in the case of kinetic resolution, detergent and food applications. When bi-logistic, the built-in algorithm decomposed the growth trajectory into two sub-trajectories. The software made a first guess at parameters, which were then refined by making subsequent fits. The grey region represents the statistical confidence of the estimated parameters. (1) and (2) were placed at the midpoint of each sub-trajectory [12].

## Results and Discussion

The patent search has retrieved 671 documents directed to kinetic resolution, 456 to detergent production, 165 to biodiesel production, and 544 to food and feed production. As to the scientific publications, 1352, 117, 439, and 315 documents have been respectively found for each application of lipases, which will be discussed in detail below.

### Kinetic Resolution

The use of enzymes for biocatalysis was long ago recognized by the industrial sector as interesting substitutes for the conventional chemical catalysts [13]. In the context of fine chemicals, the main application of enzymes as biocatalysts is in kinetic resolution to prepare enantiopure compounds, and lipases have been one of the best studied and most industrially applied enzymes [14]-[15].

The interest in lipases as biocatalysts for resolving racemic mixtures seems to have started in the 80’s, both in terms of scientific research and patent filings. It was in the same period that important results on enzymatic catalysis carried out in organic media were being published [16].

**Fig 1:**
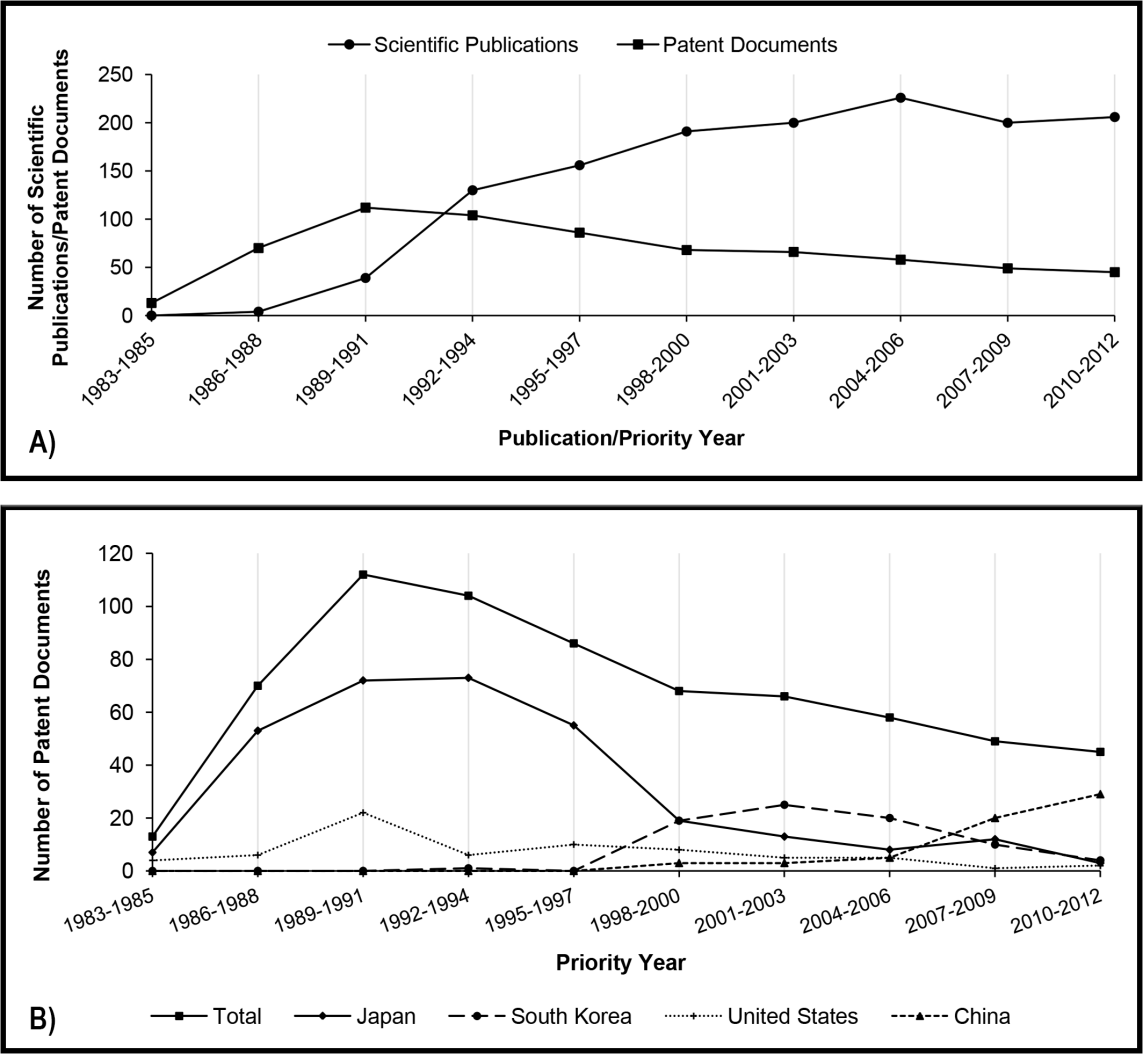
A) Total number of scientific publications and patent documents related to kinetic resolution per priority year and publication year (triennium), respectively. B) Number of patent documents related to kinetic resolution divided by the main countries of the first priority.

In the early years of analysis, the number of patent documents exceeded the scientific publications, a situation that lasted until the beginning of the 90s (Fig 1A). From this decade to the end of the study period, each indicator seems to follow a different trend: while patent filings decrease, the number of scientific publications grows. Such tendency may be explained by analyzing the assignees and authors of these documents.

It can be observed in Fig 1B that in the first two decades of study Japan was almost the only country filing patent applications directed to the use of lipases in kinetic resolution. In this period, the Japanese chemical industry was one of the triad controlling the global chemical market (along with the United States and Western Europe) [17], and the firms were massively investing in new technologies [18]. Since R&D efforts were basically made by the Japanese private sector, whilst the country lacked researches in this area, it is not surprising that the number of patents were much greater than scientific publications [19]. Japanese Corporations Chisso (current Japan New Chisso), the Mitsubishi group, Sumitomo Chemicals, Amano Enzymes, Daicel Chemical and Kawaken Fine Chemicals are within the top 10 assignees (Table 3).

**Table 3:** Top 10 patent assignees of the patent documents related to lipase and kinetic resolution having the first priority between 1983 and 2012.

Assignee	Country	Number of patent families
Chisso Corportation	JP	39
Mitsubishi Group	JP	26
Enzytech Co.	KR	21
Basf SE	DE	20
Sumitomo Chemical Co.	JP	18
Zhejiang University	CN	12
Amano Enzyme Inc.	JP	11
Bayer AG	DE	11
Daicel Chemical Industry	JP	11
Kawaken Fine Chemicals Co.	JP	10

The 1990s (and, in the opinion of some authors, the 2000s as well) are known as the “lost decade”, when the Japan’s economic bubble burst, and its chemical industry was hit hard [20]. The country has not yet been able to rebuild its chemical industry to its former glory, and the result is clearly observed in the precipitous decline in the number of patent filings.

On the other hand, the Chinese growth in the last decade is remarkable. In the last triennium (2010–2012) Chinese participation in the publication of scientific researches and file of patent application corresponded nearly to 20% and 65%, respectively. Nevertheless, unlike the other Asian countries, Chinese R&D developments in this area are mainly supported by the government sector and most of the two indicators are authored by universities. Attention should be drawn to the fact that almost all these patent documents have been filed only in China, which is in keeping with Chinese intentions to promote indigenous innovations [21].

In Fig 1A, it can be verified that the number of patent filings involving lipases for kinetic resolution is gradually decreasing in the 30 years of study. In order to compare such result with another biocatalyst, a new search was performed using the same strategy, but replacing the term “lipase” with “dehydrogenase”. Dehydrogenases are enzymes used in asymmetric synthesis; however, their industrial application may be limited by the need of using expensive cofactors [22–24].

**Fig 2:**
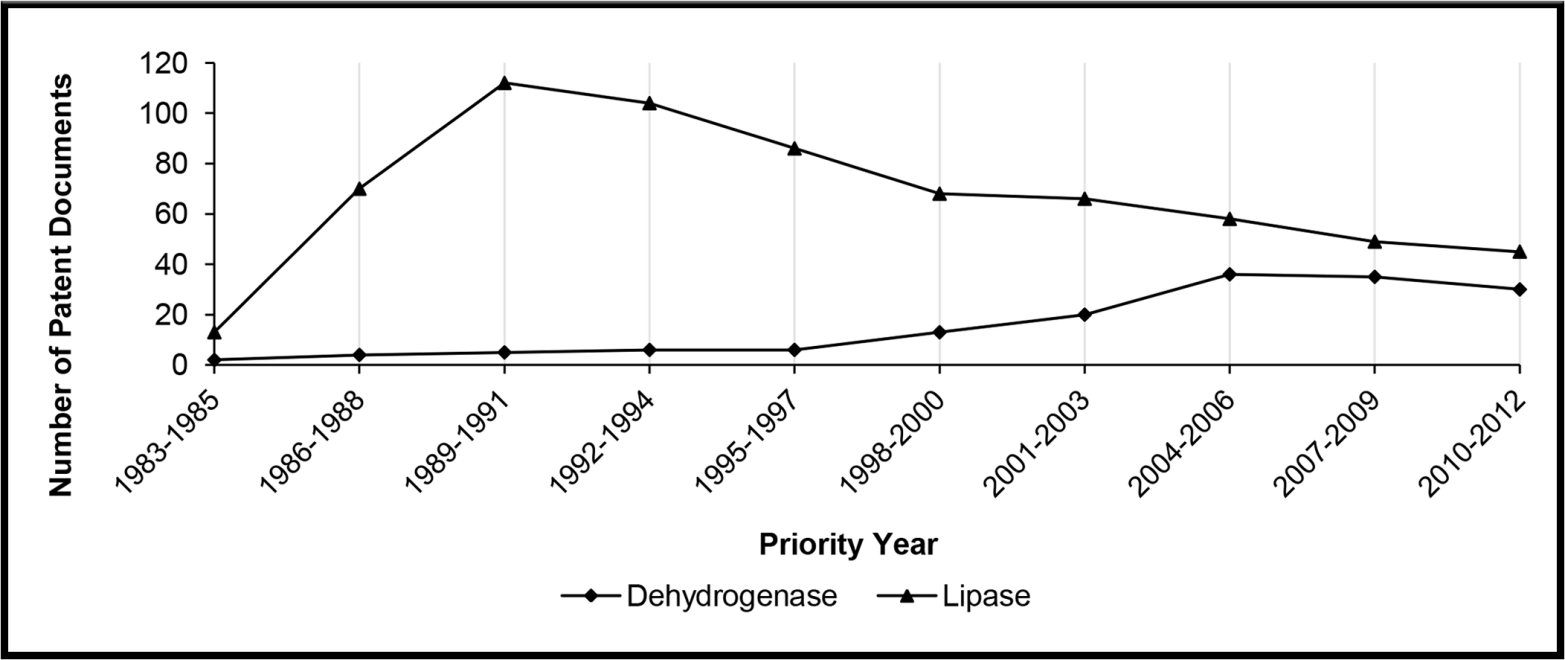
Comparison between patent documents directed to kinetic resolution using lipases and dehydrogenases as biocatalysts.

Fig 2 clearly shows that the interest in using lipases for biocatalytic resolution is older than dehydrogenases. As stated in [14], published in 1999, “hydrolases are by far the most prominent group of enzymes used in production of fine chemicals by biocatalyst resolution.” The highest number of patent filings related to lipases was reached in the third triennium (1989–1991), and it has decreased since then. In contrast, the number of filings referring to dehydrogenases was much lower up to the eighth triennium (2004–2006), but it seems to be increasing probably due to developments in researches related to cofactor recycling [25].

Other relevant information extracted from the patent documents is related to the subject matter disclosed by them. 92% of the documents mainly disclose processes for resolving racemic mixtures using lipases as biocatalysts. In contrast, only 8% are directed to the biocatalyst itself (for instance, lipases with improved characteristics, genes encoding enzymes with lipase activity, microorganisms expressing such enzymes, and method for immobilizing lipases).

By carefully reading the title and abstract of the 56 documents included in the second group, it was possible to divide them into five subgroups depending on the source of the lipase/gene disclosed, i.e., bacteria (19 documents), filamentous fungi (2 documents), yeasts (9 documents), insects (1 document) or porcine pancreas (1 document). It is worth noting that such division was made based on the disclosure provided in the title and abstract of the documents, and not all of them include such information.

Lipases also produce enantiomerically pure compounds by desymmetrization of prochiral and *meso* compounds. According to recent literature, this would be the most adequate method for obtaining enantiopure molecules since a potential 100% yield can be attained, in contrast to the maximum yield of 50% obtained by kinetic resolution processes–due to the fact that only half of the starting material is used [26–28].

Despite the theoretical advantages, the patent and literature searches related to lipases and desymmetrization have shown that kinetic resolution is still more relevant in the organic synthesis field. Scientific publications directed to the application of lipases in desymmetrization processes represent only about 10% of the studies published for lipases in kinetic resolution processes from 1983 to 2012. In what concerns patent documents, the number found for desymmetrization is not even close to that found for kinetic resolution. For example, while 216 documents were retrieved in a simple search combining “lipase? and ‘kinetic resolution’”, only 4, 9 and 8 documents were identified in the searches “lipase? and desymm*”, “lipase? and meso” and “lipase? and prochiral*”, respectively.

It should be highlighted that all the searches conducted in this study recovered patent documents containing the selected keywords in the title and/or abstract. Therefore, they are not intended for identification of all documents filed belonging to a certain field, but for the mapping of a technological field, and comparison between different fields.

### Production of Detergent Formulations:

**Fig 3:**
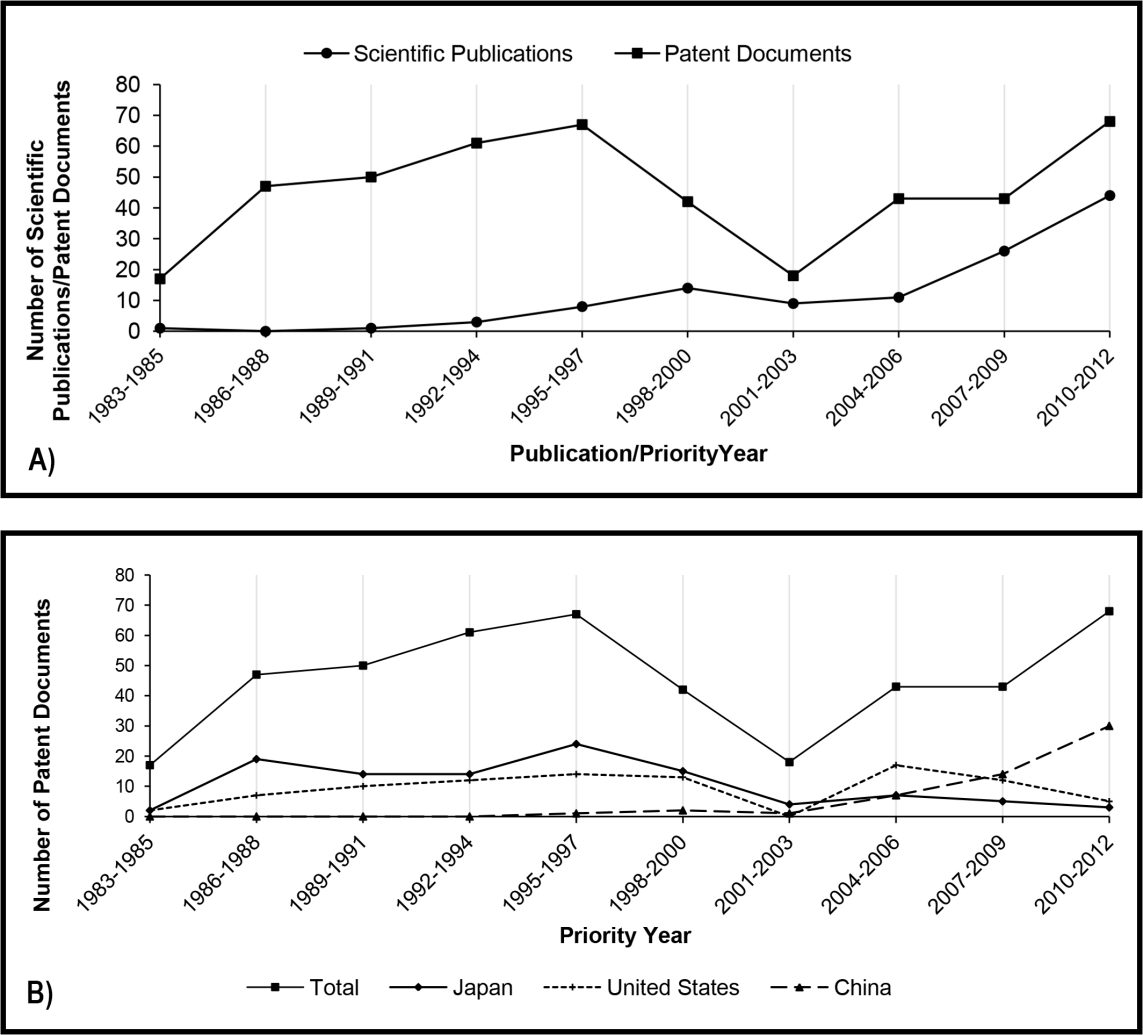
A) Total number of scientific publications and patent documents related to detergent production per priority year and publication year (triennium), respectively. B) Number of patent documents related to detergent production divided by the main countries of the first priority.

The history of the number of patent filings related to the use of lipases in detergent formulations may be divided into three distinct phases. Fig 3A shows a growth from the first to the fifth triennium, followed by a decrease up to the seventh triennium and a new increase over the last nine years analyzed.

The growth phase observed in the 80s and 90s coincided with the beginning of the use of lipases in the production of detergents: the first lipase with commercial success in this sector was Lipolase launched on the market in 1988 by Novo Nordisk A/S [29]. After that, other commercially successful lipases were also launched in the 90s, such as Lumafast and Lipomax, by Genencor [30].

Although taking into account that patent filings/publications cannot be directly correlated to market launch due to time lag [31], one possible explanation for the sharp fall observed in the second phase is the global enzyme demand commented on Li et al., 2012 [32]. The detergent industry suffered from declining sales as a result of pricing pressures at the beginning of the XXI century. Probably due to the search for new and more effective detergent compositions and enzymes, the number of both patent filings and scientific publications in this sector started growing again.

Such growth in the last three trienniums is largely attributed to the participation of China (Fig 3B). The country, together with the rest of Asia and the Pacific, was the second in enzyme demand in 2012, and future strong gains in laundry detergent market are expected to be a result from the growth in such regions [9]. Similarly to what was verified for kinetic resolution, most of these Chinese documents were filed only in China, by universities and institutes. In general, universities, institutes and foundations are assignees of no more than 10% of the patent documents related to lipases in detergent formulations, the main assignees being leading companies in the sector such as Procter & Gamble, Unilever, Lion, Novozymes, Henkel and Kao (Table 4). The household care business of Novozymes, a biotechnology company that has over 60% of the global market share for laundry detergent enzymes, grew 4% in 2014 and now represents 35% of the company’s turnover [33].

**Table 4:** Top 10 patent assignees of the patent documents related to lipase and detergent production having the first priority between 1983 and 2012.

Assignee	Country	Number of patent families
Procter & Gamble	US	72
Unilever	GB/NL	42
Lion Corporation	JP	33
Novozymes	DK	33
Novo Nordisk	DK	26
Henkel	DE	20
Kao Corporation	JP	15
Genencor	US	10
Clorox	US	8
Showa Denko	JP	8

**Fig 4:**
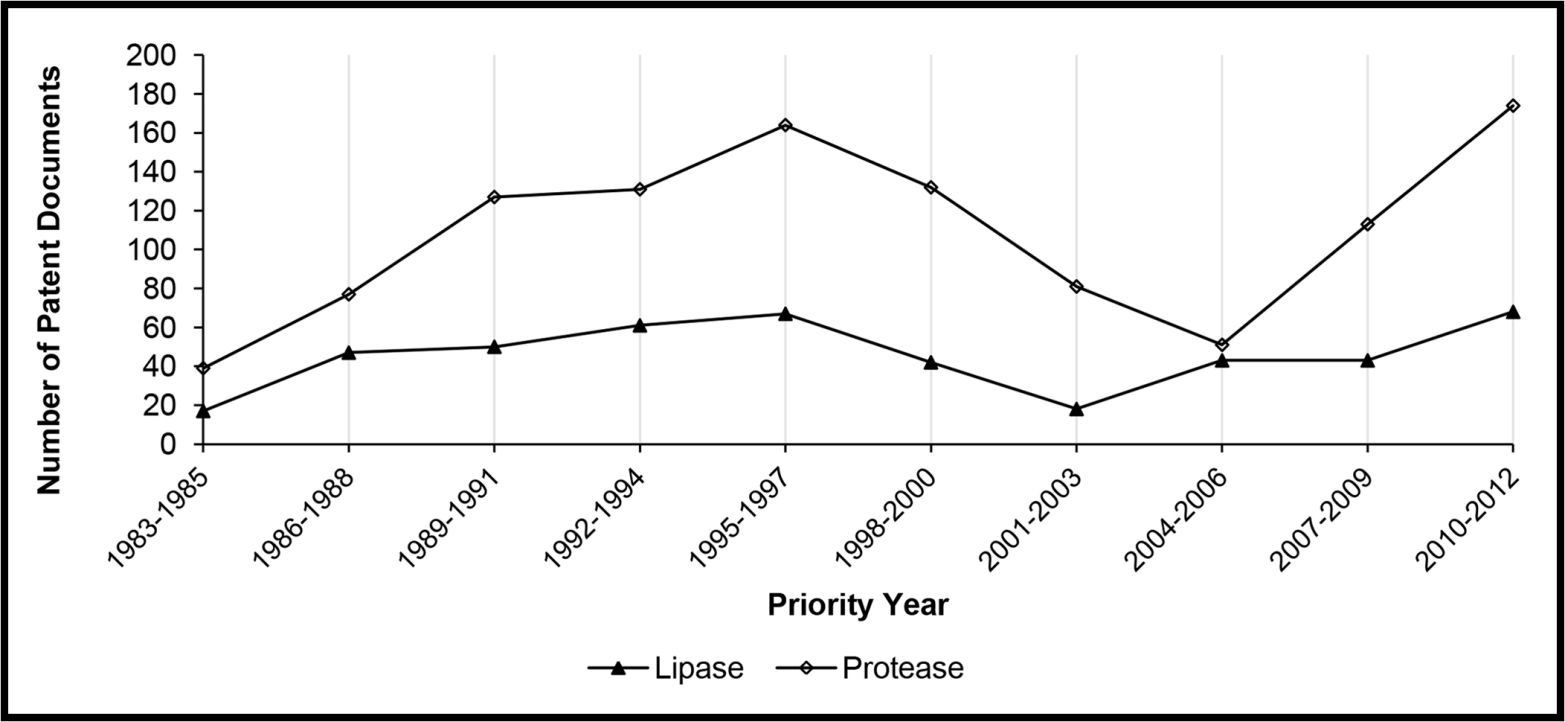
Comparison between patent documents directed to the use of lipases and proteases for producing detergents.

The profile found for the application of lipases is compared to proteases in Fig 4. A search was performed using the same strategy, but replacing the term “lipase” with “protease”. The same three phases can be observed in the history of patent filings related to the application of proteases in detergent formulations, showing that the first period of growth, the crisis and subsequent growth recovery are common to the two most used enzymes in the detergent industry.

The reading of the title and abstract of the patent documents showed that 70% of them refer to detergent formulation containing a lipase as one of the components, while 30% are directed to the biocatalyst itself. Among the documents included in the latter group, the main new features disclosed for the lipase are pH stability, temperature stability (low- and high-temperature lipases), solvent resistance and stability to proteolytic digestion. The majority of the lipases/genes are of bacterial origin (64 documents), although lipase/genes from filamentous fungi (13 documents), yeasts (6 documents) and porcine pancreas (1 document) are also disclosed.

### Production of Food and Feed Products:

Food technology is an important market for enzymes. In 2014, the Food & Beverage industry was the second most important market for Novozymes’ products, accounting for 26% of the Danish company’s sales. The two most significant contributors to the 4% increase in Novozymes’ sales to this sector in 2014 were the use of enzymes for baking purposes and for the production of healthy food, two important sectors for application of lipases [33].

Lipases find several other uses in food technology, *inter alia* in flavor development, manufacture of dairy products, production of butter and milk equivalents, processing of meat and fish, and animal feed. Due to its broad field of action, this group of enzymes is considered to have a large untapped potential in the food preparation sector [7].

**Fig 5:**
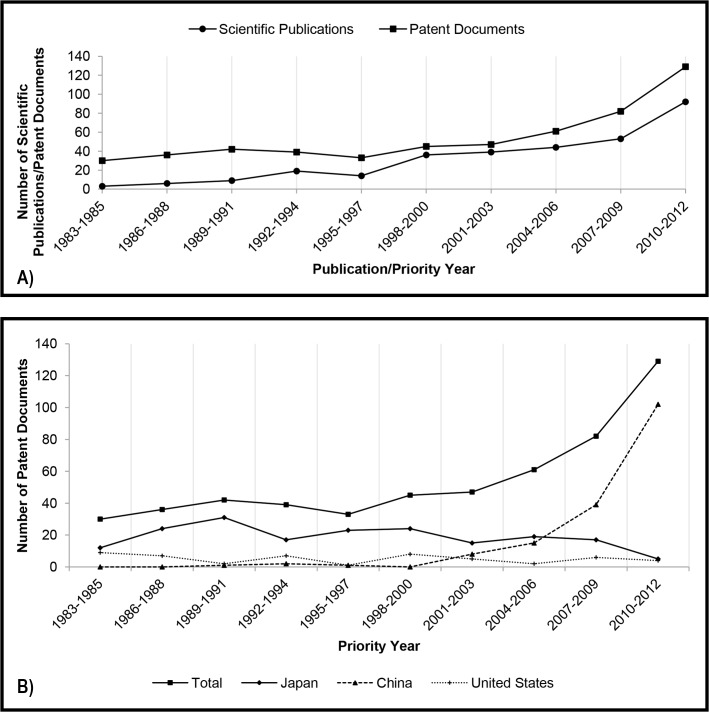
A) Total number of scientific publications and patent documents related to food and feed production per priority year and publication year (triennium), respectively. B) Number of patent documents related to food and feed production divided by the main countries of the first priority.

In the first twenty years analyzed in this study, the number of patent documents filed annually varied from about thirty to forty-five patent families. The number of scientific publications was considerably smaller in the first five trienniums; however, it seems that the application of lipases in food technology started to capture researches’ attention in the late 1990s (Fig 5A).

The growth in the filing numbers of patent documents related to lipase and food and feed production was clearly leaded by Japan until the early 2000s. After that, Chinese participation is gaining importance, and China has become the priority country of the majority of the patent families since the ninth triennium (2007–2009). An impressive 79% of the patent families filed in the tenth triennium (2010–2012) have Chinese priority (Fig 5B).

Chinese growth in recent years is so noticeable that the first in the ranking of top ten patent assignees is a Chinese university. Except for Jiangnan University all the other assignees are companies, such as Nestlé and Kraft Foods, two giants of the food industry. The biotechnology companies Novozymes and DSM are also found in the ranking, owning 12 and 7 patent families, respectively (Table 5). In general, universities, institutes and foundations have little contribution to the filing of patent documents related to food technology, accounting for 17% of the families unveiled in the patent search.

**Table 5:** Top 10 patent assignees of the patent documents related to lipase and food and feed production having the first priority between 1983 and 2012.

Assignee	Country	Number of patent families
Jiangnan University	CN	16
Novozymes	DK	12
Nestlé	CH	11
Fuji Oil	JP	10
T. Hasegawa	JP	10
Nestec	CH	7
Kraft Foods	US	7
Shenzhen Leveking Biology Engineering	CN	7
DSM	NL	7
Nisshin Oil Mills	JP	6

The documents retrieved in the patent search were divided in two groups depending on the subject matter disclosed therein. Such division was performed based on the reading of the title and abstract of the documents. Approximately 11% of them were classified as referring to the biocatalyst itself, i.e. documents included in the molecular biology field dealing with a lipase, its gene or production method, and the like. The remaining 89% were related to a food, feed or beverage product prepared by using a lipase or a process for preparing such products.

The first group was then analyzed with relation to the source of the lipase/lipase gene. 42 out of the 58 documents had such piece of information in their title or abstract. In 16 of them the lipase source was a bacterium; in 15, a filamentous fungus; in 7, a mammal; in 3, a yeast and in 3, an *Archaea*. Two of them were related both to a bacterium and an *Archaea*.

### Production of Biodiesel

**Fig 6:**
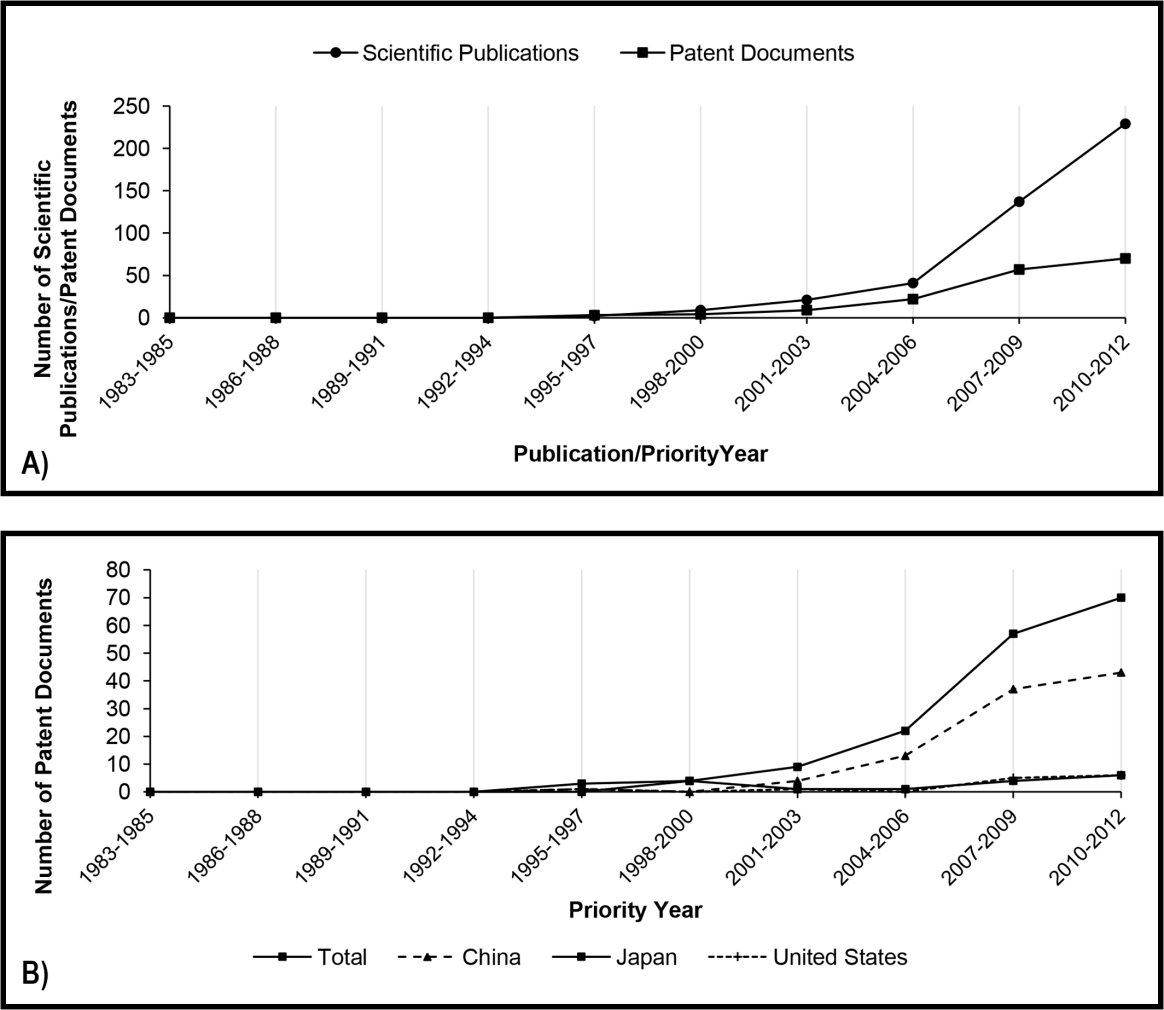
A) Total number of scientific publications and patent documents related to biodiesel production per priority year and publication year (triennium), respectively. B) Number of patent documents related to biodiesel production divided by the main countries of the first priority.

Among the four applications analyzed in the present study, the use of lipases for producing biodiesel is clearly the most recent one. Both scientific publications and patent filings started gaining importance in the beginning of 2000s. Differently from the two applications examined previously the number of patent filings was lower than scientific publications throughout the analysis period (Fig 6A). This result may be explained by the fact that the enzymatic transesterification process is a technology mainly developed by academic research. 69% of the patent documents were filed by universities, institutes and foundations, and six from the top ten assignees are universities (Table 6). The use of lipases as biocatalysts for biodiesel production is not yet a method widely used in industry due to the higher costs of the enzyme when compared to the alkaline catalyst. [11] has estimated enzyme (Novozym 435) and NaOH prices as about US$ 0.14 and US$ 0.006 per kilogram of ester, respectively.

**Table 6:** Top 10 patent assignees of the patent documents related to lipase and biodiesel production having the first priority between 1983 and 2012.

Assignee	Country	Number of patent families
Tsinghua University	CN	14
Kansai Chemical Engineering	JP	5
Beijing University of Chemical Technology	CN	4
Bio Energy	JP	4
Huazhong University of Science & Technology	CN	4
TransBiodiesel	IL	4
Shejiang University	CN	4
Cognis	DE	3
Henan Agricultural University	CN	3
Chosun University	KR	3

The first attempts for producing biodiesel via enzymatic transesterification in industrial scale were made in the beginning of the 2000s by Chinese companies. China is a country which is a reference in this field and has a significant participation both in the filing of patents (Fig 6B) and publication of scientific papers. In 2006, Hainabaichuan Co. Ltd established a plant with 20,000 tons of biodiesel production capacity per year using technology of the Tsinghua University, employing commercial lipase Novozym 435 and waste palm oil. This plant was doubled to 40,000 tons of biodiesel per year in 2008. In 2007, Lvming Co. Ltd., by using technology developed by the University of Beijing, immobilized *Candida sp*. lipase and waste cooking oil as feedstock, opened its plant with capacity of 10,000 tons of biodiesel per year [34–35].

Another country that has stood out in this area is the United States. From 2012 to 2014, three plants on a commercial scale were launched by North American companies. In 2012, Piedmont Biofuels announced the launch of its plant for production of biodiesel through a new technology (FAeSTER), with collaboration from Novozymes [34]. In 2013, Viesel Fuel LLC announced the start of its production of biodiesel by the transesterification process also developed in collaboration with Novozymes, which has capacity to produce 7 million gallons annually [36]. A year later, Blue Sun Biodiesel established its plant with production capacity of 30 million gallons per year, also using enzyme technology from Novozymes [37].

In relation to the content of the patent documents, the reading of the title and abstract showed that 57% of them are directed to new methods of producing biodiesel by using lipases, while 32% are related to the biocatalyst itself, i.e. lipases, their genes and microorganisms producing them, capable of being used for producing biodiesel. The remaining 11% disclose inventions such equipments for producing biodiesel by using lipases as biocatalysts (7%) and lipases used as one component of a fuel additive (4%). An important feature observed in these documents is the immobilization of the biocatalyst, which appears in 27% of the titles and abstracts analyzed.

When considering the group including the documents directed to the biocatalyst, 26 of them include in the title and abstract information regarding the origin of the lipase/gene; bacteria (13 documents), filamentous fungi (7 documents), yeasts (4 documents), algae (1 document) or plant (1 document).

### Technological Forecasting

There are several examples in the literature wherein S-curves have been used for technological forecasting purposes [38–41]. Such method of modeling technology life cycle (TLC) is based on the assumption that technology growth trajectory follows a logistic model, that is, a system growths exponentially until reach an upper limit (or carrying capacity) inherent to the system. The logistic function is based on three parameters: the saturation level or limit, the midpoint (when the curve reaches half of the saturation level), and the characteristic duration (time required for the trajectory to grow from 10% to 90% of the limit). [42].

There are situations in which growth processes occur in pulses, which may be sequential or simultaneous, and the growth trajectory may be calculated as the sum of multiple pulses. In cases in which two growth phases are observed, the system is called bi-logistic, and the bi-logistic model is useful to model systems exhibiting complex growth that do not fit in the simple logistic function [42].

One S-curve approach plots cumulative patent data over time to monitor the attractiveness of a technology for investment [43]. Patenting activity is a good indicator of technological performance as it measures both the technological development and the market diffusion of a certain technology [44].

One drawback of this method is that the validity of the results is very sensitive to the amount of data [39]. Therefore, when a technology is in its early growth stage, the projected trajectory may greatly deviate from the actual future growth [38].

**Fig 7:**
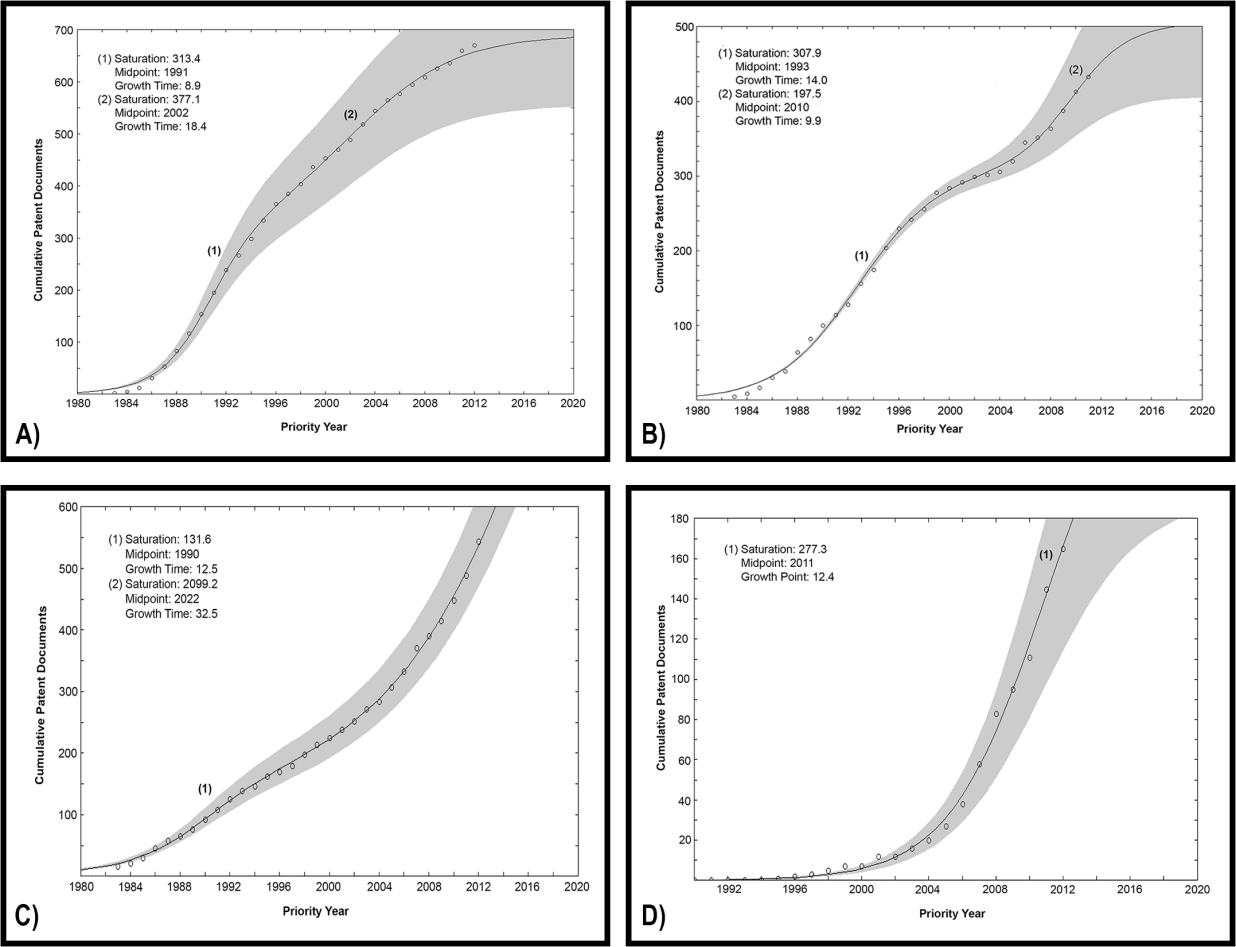
Cumulative patent data fitted into S-shaped curves using Loglet Lab 2 Software. Growth trajectories of lipase and A) kinetic resolution technology; B) detergent production technology; C) food and feed production technology; and D) biodiesel production technology.

In the case of racemic resolution (Fig 7A), the first growth pulse overlaps the Japanese progress in filing patent applications related to such technology. It started in 1986, reached the midpoint in 1991 and matured in 1995. The above stages respectively correspond to 10%, 50% and 90% of the growth limit. The second growth pulse started in 1992, possibly associated with the Japanese crisis and participation of other players in the filing of patent applications. The midpoint and mature year were respectively reached in 2002 and 2012.

According to these results, the technology of applying lipases as biocatalysts for kinetic resolution would have already finished its maturity stage, thereby currently being in the last phase of an industrial life cycle, i.e., the saturation stage. Nevertheless, it is often mentioned in the literature that organic synthesis is one of the most promising industrial application of enzymes [45–47]. In addition, a recent report on enzyme market indicates that strong gains are forecast for lipases acting as biocatalysts for fine and specialty applications [9].

In fact, lipases are one of the most important enzymes in the context of industrial biocatalysis. There are some examples of well-established processes for production of fine chemicals using these enzymes, such as the synthesis of Lyrica (pregabalin) by Pfizer, which applies the enzyme Lipolase, a lipase marketed by Novozymes A/S. However, the examples of commercial scale application of biocatalysts are still beneath the expectations, and the potential of biocatalysis has not yet been reached [47–49].

Some developments are expected with the so-called “third wave of biocatalysis”, and the present work showed that researches in this area have been conducted by the academic sector, as there is a considerable number of scientific works being published.

With the progress of tailoring new biocatalysts so as to adapt them to industry conditions and render them cost competitive with chemical catalysts, the advantages of the enzymes may probably make same the first choice in industrial processes for the production of e.g. fine chemicals.

In this context, it is not impossible to expect that a new growth phase will start in the next few years, mainly if considered the increasing participation of countries such as China and India in producing knowledge in the biotechnology sector. In this case, the curve shown in Fig 7A will no longer be bi-logistic, as a third period of logistic growth will appear.

On the other hand, it is important to consider that major pharmaceutical companies are shifting their R&D expenditures towards biologics. The number of new biologic drugs being searched by such companies is now almost equivalent to that of small molecule candidates [50]. A study comparing newly launched small molecules and biologics during a ten years period has shown a ratio close to one-to-one in 2008 [51].

The global demand for biopharmaceuticals is growing fast, and they currently account for approximately 20% of the total pharmaceutical market. The annual growth of biopharmaceutical market of more than eight percent is double compared to that of conventional pharma [50], [52]. Hence, it is not surprising that pharmaceutical companies are more and more focusing on the development of biologics, which may be negatively affecting the use of enzymes for the preparation of small molecular drugs.

The production of enantiomerically pure amines via kinetic resolution processes is a key use of lipases [53]. Considering that aminotransferases/transaminases are other enzymes capable of producing these compounds from achiral ketones, the increasing use of the latter group could be seen as another reason for the saturation phase observed in the use of lipases in kinetic resolution. The literature states that asymmetric biocatalytic production of chiral amines is rarely used, but focus has been turned to asymmetric synthesis as they are more efficient processes [54]. Additional patent and scientific publication searches have been conducted in order to confirm this hypothesis. Indeed, it has been verified a considerable increase in the number of scientific publications in the last years related to aminotransferases/transaminases in kinetic resolution and asymmetric synthesis; however, this number is still much lower than the published works directed to lipases and kinetic resolution (68 and 206, respectively, in the triennium 2010–2012). Only 85 patent documents related to aminotransferases/transaminases have been found from 1983 to 2012. These results seem to corroborate the study of Tufvesson *et al* wherein it is said “that the state-of-the art in transaminase processes has been insufficient for successful industrial application until very recently.” [53]

The life cycle of the technology of lipases and detergent compositions also comprises two growth stages, but in this case the second pulse started growing only when the first had reached its maturity stage (Fig 7B). The first pulse started in 1986, had its midpoint in 1993 and matured in 2000. The growth stage of the second pulse began in 2005, the midpoint was reached in 2010 and its saturation stage is forecasted to initiate in 2015.

These pulses correspond to the phases previously identified in the present work. The first one covers the beginning of the technology until the decrease in the global demand that occurred in the turn of the century. The second is related to the resumption of growth of patent filings and scientific publications. If the rate of patent filings remains the same, this technology will achieve its saturation stage in 2015.

A recent report on enzyme demand states that the demand for enzymes for application in detergents and other cleaning products will increase 6.2 percent annually, reaching US$ 1.2 billion in 2017. Such growth will be mainly due to the increasing market penetration in developing economies, such as Asia/Pacific and South America, but will be only moderate in North America and Western Europe. Furthermore, the strong competition between the producers has stimulated them to introduce new and more efficient enzymes, optimized for some conditions such as low temperature [9].

Therefore, as discussed for the case of kinetic resolution, the production of new lipases with improved properties may attract the attention of the industrial sector towards the preparation of new detergent compositions.

Similarly to the two applications discussed above, the application of lipases in food technology has two growth pulses. The first one had its midpoint in 1990 and matured in 1996. The second pulse, among all the technologies analyzed, is the one that is farther from reaching the mature year. The midpoint is predicted to be reached in 2022 and the saturation phase will probably begin around 2038 (Fig 7C).

The food market is seen as a sector with high growth potential by both food manufactures and enzyme suppliers. The industry is seeking for products with higher added value and more appealing to consumers for being healthier and tasting better, which paves the way for the use of enzyme technology. Public concern about quality, safety and sustainability of food products is increasing, and healthy foods are one of the responsible for having increased sales of the enzyme suppliers in the last years [33], [55].

The application of lipases for transesterification of biodiesel is such a recent technology that its growth trajectory fits in a logistic curve (Fig 7D). According to the graph, the trajectory reached the midpoint in 2011 and its mature year will be in 2017. As disclaimed above, as the amount of data is small, the forecasted trajectory may greatly deviate from the actual future growth, and the projection is less reliable.

Table 7 summarizes the characteristics of each growth trajectory.

**Table 7:** Characteristics of the S-curves built in Loglet Lab 2 for each technology addressed in this study.

Technology	Curve Type	Midpoint (year)	Growth Time (years)	Mature Year	Midpoint (year)	Growth Time (years)	Mature Year
		(1)	(1)	(1)	(2)	(2)	(2)
Kinetic Resolution	Bi-logistic	1991	8.9	~1995	2002	18.4	~ 2012
Detergent	Bi-logistic	1993	14.0	~ 2000	2010	9.9	~ 2015
Food and Feed	Bi-logistic	1990	12.5	~ 1996	2022	32.5	~ 2038
Biodiesel*	Logistic	2011	12.4	~2017	-	-	-

Midpoint is the time when 50% of the growth limit is reached; growth time is the period for the trajectory to grow from 10% to 90% of the limit; mature year is the time when 90% of the growth limit is reached. *As the amount of data is small, the forecasted trajectory may greatly deviate from the actual future growth, and the projection is less reliable.

## Conclusion

Both scientific and technological indicators related to four strategic sectors applying lipases were analyzed. The data indicate that these enzymes are still important biocatalysts, and this result is in keeping with the latest report launched by the Freedonia Group in 2014, according to which the world demand for lipases is projected to increase 6.2% annually to US$ 345 million in 2017. Although this growth is expected to be driven by both industrial and specialty markets, the present study pointed out specificities of each of the applications analyzed.

The kinetic resolution sector, which has been firstly promoted by the Japanese chemical industries, seems to have lost its industrial dynamism. The most of the patent applications having been filed are assigned to Chinese universities and are mainly restricted to China. This sector, however, appears to be still attractive in what concerns academic researches. Although lipases are one of the most common biocatalysts used in the last decades, there are only a few examples of the application thereof in the preparation of fine chemicals on a commercial scale. Although the perspectives of the industrial biotechnology are very good, its actual application is far from its high potential. Future developments made by the academy, essentially related to new lipases based on direct evolution, are expected to overcome the technical challenges and boost the sector.

After a crisis period, the detergent formulation sector seems to have been in expansion after the 2000’s. Considering the small number of scientific publications related to lipase and detergent formulation and the also small participation of the academy in the filing of patent applications, the historical importance of the industrial R&D in this sector is highlighted. In the thirty years analyzed, the main assignees of patent documents were big consumer goods companies. In the last triennium, however, the participation of Chinese universities was the main responsible for the new increase in the numbers of patents and scientific publications. The third wave of biocatalysis, which is the one currently in force, is expected to assist on the predicted growing of the sector with the development of new enzymes having improved properties.

Food technology is an important sector for the application of enzymes. It has increasingly been attracting the attention of researchers and applicants in the last years, and considerable growth is still expected for this industry. Increasing public concern about health issues is one of the factors driving such growth. Chinese market is a challenge for the major enzyme suppliers, and attention should be drawn to the fact that China is the first priority country of 79% of the patent families filed in the last triennium analyzed in this study.

As to the application of lipases for producing biodiesel, clearly the younger sector of the four sectors analyzed, it seems to be in a broad expansion as well. As observed in this study, the major advances and researches made in this sector has been carried out by universities, institutes and foundations, and the first attempts to produce biodiesel by enzymatic transesterification in commercial scale date back to the early 2000s. The actual industrial use of these biocatalysts represents only a great potential, but once the enzymatic transesterification is cost competitive with the alkaline method, the advantages of the use of lipases will probably make them the preferred type of catalyst.
